# Embedded Living HER2+ Cells in a 3D Gelatin–Alginate Hydrogel as an In Vitro Model for Immunotherapy Delivery for Breast Cancer

**DOI:** 10.3390/polym15183726

**Published:** 2023-09-11

**Authors:** G. Tonantzin De Dios-Figueroa, Janette del Rocío Aguilera-Márquez, Lorena García-Uriostegui, Rodolfo Hernández-Gutiérrez, Tanya A. Camacho-Villegas, Pavel H. Lugo-Fabres

**Affiliations:** 1 Unidad de Biotecnología Médica y Farmacéutica, Centro de Investigación y Asistencia en Tecnología y Diseño del Estado de Jalisco (CIATEJ), Guadalajara 44270, Jalisco, Mexico; gdedios_al@ciatej.edu.mx (G.T.D.D.-F.); jaaguilera_al@ciatej.edu.mx (J.d.R.A.-M.); rhgutierrez@ciatej.mx (R.H.-G.); 2 CONAHCYT—Departamento de Madera, Celulosa y Papel, Universidad de Guadalajara (UDG), Guadalajara 44100, Jalisco, Mexico; lgarciaur@conacyt.mx; 3 CONAHCYT—Unidad de Biotecnología Médica y Farmacéutica, Centro de Investigación y Asistencia en Tecnología y Diseño del Estado de Jalisco (CIATEJ), Guadalajara 44270, Jalisco, Mexico; tcamacho@ciatej.mx

**Keywords:** hydrogel, three-dimensional (3D) cell culture, living embedded cells, breast cancer, HER2+

## Abstract

Epidermal growth factor receptor 2 (HER2) is the second target molecule most commonly used in breast cancer treatment. Both recurrence and metastasis are still deadly for HER2+ breast cancer patients. Hydrogels can be an option for developing three-dimensional (3D) cell culture systems that resemble tumor features better than monolayer cultures and could be used for preclinical screening for new biotherapeutics. Biopolymers (gelatin and alginate) were used to develop a hydrogel capable of encapsulating living HER2+ breast cancer cells BT-474/GFP. The hydrogel was physicochemically characterized, and the viability of embedded cells was evaluated. The hydrogel developed had suitable physical properties, with swelling of 38% of its original mass at 20 h capacity and pore sizes between 20 and 125 µm that allowed cells to maintain their morphology in a 3D environment, in addition to being biocompatible and preserving 90% of cell viability at 10 days. Furthermore, encapsulated BT-474/GFP cells maintained HER2 expression that could be detected by the Trastuzumab-fluorescent antibody, so this hydrogel could be used to evaluate new HER2-targeted therapies.

## 1. Introduction

Breast cancer is the uncontrolled growth of breast cells [1], which can be epithelial cells (that surround breast ducts or lobules) or stromal cells [2]. In 2020, there were about 2.26 million new cases and almost 685 thousand deaths globally due to this disease [3]. Although women at any age after puberty can suffer breast cancer, it is more frequent in women between 50 and 69 years, and only 7–10% of women under 40 years are positive for this disease [4]. 

One of the treatment options for breast cancer is targeted therapy, which refers to all those treatments specific to a molecular target, such as cell receptors or proteins involved in tumor processes. The receptor most commonly used in breast cancer treatment is epidermal growth factor receptor 2 (HER2) [5,6]. Overexpression of the HER2 receptor is present in 20–30% of breast cancer tumors, including cancers classified as HER2 positive (HER2+) and Luminal B cancer. HER2 is a 185 kDa protein that can form homodimers or heterodimers with other HER receptors. When dimerization occurs, tyrosine residues in the intracellular domain are phosphorylated, activating signal pathways such as mitogenic protein kinase (MAPK) and phosphatidylinositol 3-kinase (PI3K). Different dimeric combinations produce different intracellular signaling cascades [2,7]. Activation of HER2 is associated with excessive cell division and tumor formation. HER2+ cancers are more aggressive, with a higher risk of metastasis, and patients have significantly shorter survival [2]. HER2 is also overexpressed in other neoplasms such as the bladder, uterus, ovary, stomach, biliary tract, and pancreas cancer [8].

Treatments directed at HER2 are currently on the market for its intracellular and extracellular domains. Trastuzumab (used as adjuvant therapy in metastatic cancer), Pertuzumab (used as adjuvant therapy in metastatic cancer), Lapatinib (used in metastatic cancer), Trastuzumab emtansine (used in metastatic cancer), and Neratinib (used in adjuvant therapy) [9] are some of them. Nevertheless, a significant fraction of HER2-overexpressing tumors do not respond to chemo-drug therapies or eventually acquire resistance. The mechanisms by which this resistance is acquired remain unclear. Furthermore, tumor reduction or surgical remotion does not mean the disease has been cured. Therefore, identifying and investigating more effective therapies for anti-HER2 is an urgent medical need [10] associated with developing animal-free preclinical models that allow for faster clinical translation. 

Three-dimensional (3D) cell culture systems resemble extracellular matrix (ECM) structures, besides mimicking cell–cell and cell–ECM interactions and giving valuable information about in vivo cell responses [11]. The 3D cell culture systems are essential in cancer research because they can imitate heterogeneous hypoxic conditions and tumor microenvironment (TME) characteristics [12,13]. Furthermore, cell–cell and cell–ECM interactions influence cancer proliferation, resistance, and metastasis, underlining the relevance of the 3D model having essential characteristics that allow the maintenance and analysis of these interactions.

Hydrogels are polymer networks highlighted as 3D cell culture platforms because of their swelling capacity and tunable physicochemical properties. Hydrogel properties can be controlled by material composition and cross-linking methods [14]. Gelatin is a biocompatible and biodegradable polymer with biomedical applications. However, gelatin has a fast degradation rate and poor mechanical properties; therefore, other materials, such as alginate or improving its cross-linking techniques are required [15]. Gelatin–polysaccharides hybrid hydrogels resemble the native conditions of the ECM and improve the stability of the scaffolds [16]. 

Here, we developed a hybrid gelatin–alginate hydrogel that is biocompatible with embedded HER2+ breast cancer cells. Also, embedded living BT-474/GFP cells maintained HER2 receptor expression. The hydrogel microstructure permitted cells to grow in a three-dimensional morphology and moreover, allowed the interaction between an anti-HER2 monoclonal antibody and the HER2 receptor. Hence, this hydrogel could be used to evaluate novel therapies directed at HER2 receptors in a tissue-like context.

## 2. Materials and Methods

### 2.1. Biopolymer Syntesis and Characterization

#### 2.1.1. Hydrogel Fabrication (Biopolymer Mix) without Cells

**Figure 1:**
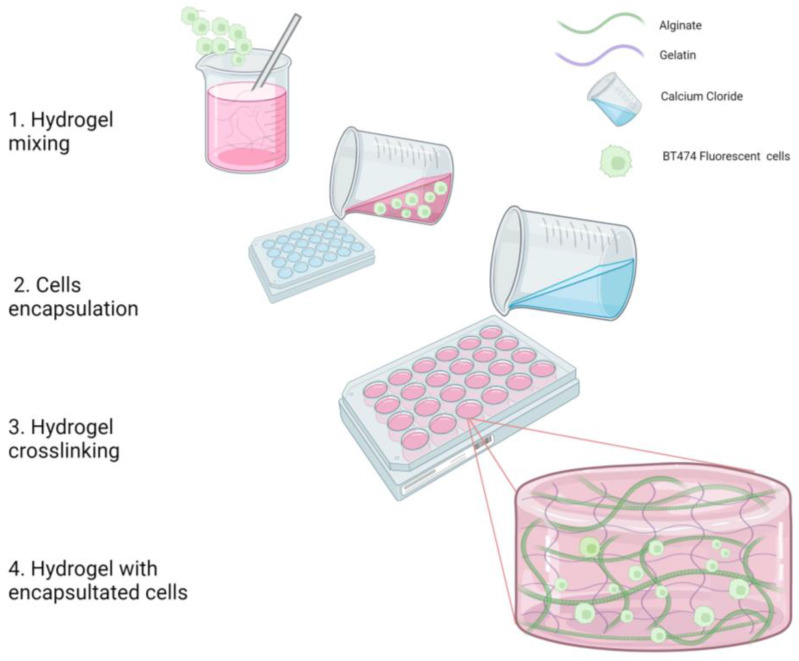
Cell encapsulation process. The schematic represents the process to obtain a fully functional hydrogel with viable embedded cells.

Alginate (food grade) was weighed in a 15 mL conical tube and dissolved in a 3 mL Hanks buffer to obtain a 20% stock solution. This alginate solution was mixed with a spatula and autoclaved. Gelatin (230 bloom) was sterilized for 30 min in UVA/UVB light inside a laminar flow hood. Then, a 20% gelatin stock solution was prepared in Dulbecco’s Modified Eagle’s Medium/Nutrient Mix F12 medium (DMEM/F-12, Sigma-Aldrich, Burlington, MA, USA). The alginate solution was dissolved in a flow hood with a non-supplemented DMEM/F-12 medium and the gelatin solution, then mixed with a sterile spatula. Later, it was heated at 60 °C for 5 min and placed in an ultrasonic sonicator until bubbles were eliminated. This gelatin, alginate, and culture medium solution was called “biopolymer mix or hydrogel”. The hydrogel was placed in a sterile cylindrical silicone mold (1 cm × 1 cm), and then incubated for 1 h at 4 °C. The final composition of the hydrogel was 7.5% gelatin (*v*/*v*), 3.75% alginate (*v*/*v*), and 0.6 mL of Hank’s solution per milliliter of culture medium (*v*/*v*). For the cross-linking process, the biopolymer mix was placed in a 150 mM CaCl_2_ solution and then incubated for 30 min at room temperature (Figure 1). After the cross-linking process, the hydrogels were mold-released, removing all CaCl_2_, and the hydrogels were washed three times with sterile distilled water and once with 1× PBS. These cell-free hydrogels are used as controls.

#### 2.1.2. Cells Encapsulation in the Biopolymer Mix 

The BT-474/GFP cell line was obtained from (AntiCancer Inc., San Diego, CA, USA). This cell line constitutively expresses GFP protein since it was transfected with a GFP vector and also expresses the HER2 membrane receptor. Initially, the cells grew in DMEM/F-12-supplemented medium (10% of FBS, 1X of antibiotic/antimycotic solution) and were cultured in standard conditions until 80% confluence. Then, the culture was trypsinized for 3 min (1× trypsin-EDTA, GIBCO) and resuspended in medium, and cell viability was determined by trypan blue. 

For the hydrogel fabrication with embedded cells, we proceeded with the same biopolymer mix solution proportion described in Section 2.1.1, with a few modifications: before the hydrogel cross-linking process with CaCl_2,_ a total of 500,000 BT-474/GFP cells were added to the hydrogel and mixed with mild agitation in a 50 mL beaker (Figure 1). Then, the cross-linking process continues as previously described with the addition of CaCl_2,_ followed by mold release and washing steps. Then, hydrogels containing embedded cells were placed in 24-well plates; 1 mL of DMEM/F-12 supplemented culture medium was added to each well and incubated at 37 °C with an atmosphere enriched with 5% CO_2_. All experiments are conducted in triplicate under sterile conditions.

### 2.2. Swelling Capacity 

Cell-free hydrogels were made and weighed (this weight is defined as “t_0_”). After that, hydrogels were incubated in lidded containers with 20 mL of Hanks saline solution at 37 °C. Hanks solution was removed at 0, 1, 2, 3, 4, 6, 8, 20, 24, 28, and 48 h, and the hydrogels were weighed (defined as “Hydrogel Weight”). The percentage of swelling for each time was calculated with the following formula:

$$\%\;Swelling=\frac{Hydrogel\;Weight-t_{0}\;Weight}{t_{0}\;Weight}\times 100$$

### 2.3. Fourier-Transform Infrared Spectroscopy (FTIR)

Hydrogels with and without cells were made under sterile conditions and analyzed by Fourier-transform infrared spectroscopy (FTIR). The spectra were collected with a Perkin-Elmer Spectrum GX spectrometer (Perkin Elmer, Waltham, MA, USA) at room temperature in the 4000–550 cm^−1^ wavenumber range. Samples were confined in a diamond ATR accessory (PIKE Technologies, Madison, WI, USA) and recorded an average of 32 repetitive scans with a resolution of 4 cm^−1^. 

### 2.4. Hydrogel Microstructure and Pore Size

Hydrogels were analyzed by scanning electron microscopy (SEM). Hydrogel samples were frozen with liquid nitrogen and lyophilized in a freeze dryer (FreeZone 1 L, Benchtop, Labconco, Kansas City, MO, USA) with a pressure of 1 Pa at −50 °C overnight. The water content was sublimated. Then, the lyophilized hydrogels were fractured, overlaid with carbon, and analyzed by scanning electron microscopy (SEM, Tescan Mira3 LMU, Tescan, Brno, Czech Republic) with a 10 kV field emission gun at different magnifications. For pore size determination, the obtained images were analyzed with Fiji image software (ImageJ version 1.53c).

### 2.5. Hydrogel Biocompatibility and Metabolic Activity 

The biocompatibility of hydrogel with cells embedded was evaluated by a resazurin reduction assay. The basis of this assay is resazurin (with a purple color) that is reduced to a red fluorescent dye, or resurafin, by the cellular mitochondrial dehydrogenase in a proportional relationship with the living cells.

Hydrogels with embedded cells (BT-474/GFP HER2+) were incubated for 24 h at 37 °C in an atmosphere enriched with 5% CO_2_. Then, 1 mL of DMEM/F-12 full supplemented culture medium with 10× resazurin reagent were added (resazurin final concentration). Hydrogels were incubated for 24 h and protected from light. Then, 100 μL of each well was taken and transferred to a 96-well plate. The 96-well plate was read in an xMark microplate spectrophotometer at 570 nm and 600 nm. The monolayer cells are used as a resazurin reduction positive control.

For the metabolic activity assay, the equation was used. The same procedure was performed on hydrogels with embedded cells, monolayer cells, cell-free hydrogels, and empty wells as controls. This process was repeated after 1, 3, 5, and 10 days of incubation. All assays are in triplicate and interpreted according to the following formula:

$$=\frac{\varepsilon OX\text{\_}600nm\times A570nm\text{\_}tx-\varepsilon OX\text{\_}570nm\times A600nm\text{\_}tx}{\varepsilon RED\text{\_}570nm\times A600nm\text{\_}t0-\varepsilon RED\text{\_}600nm\times A570nm\text{\_}t0}$$

### 2.6. Morphology and Distribution of Embedded Cells within Hydrogel

After 48 h of incubation, hydrogels were placed in a 4% paraformaldehyde solution and kept at 4 °C for 72 h. After this, the hydrogels with embedded cells were transferred to a 30% sucrose solution in 1× PBS and incubated at 4 °C for 48 h or until hydrogel sedimentation. Hydrogels were cut longitudinally with a scalpel, and half of the hydrogel was placed on the cryostat and coated with Tissue-Tek (Sakura, Osaka, Japan). It was kept at −25 °C for 10–15 min. Sections with a thickness of 40 μm were made and placed on slides treated with 2% gelatin to promote adherence. Nuclei were stained with a Hoechst (1 μg/mL, Sigma-Aldrich) and analyzed by confocal microscope.

### 2.7. Evaluation of Genes Related to Hypoxia, Apoptosis, and HER2 Receptor

#### 2.7.1. Total RNA Extraction

Total RNA from 500,000 monolayer cell cultures (BT-474/GFP HER2+) was isolated with TRIsure (Bioline) following the manufacturer’s recommendations. The RNA pellet was resuspended in molecular biology grade water, quantified by a NanoDrop 2000 (Thermo Scientific, Waltham, MA, USA), and analyzed on a 1% agarose gel to verify its integrity. 

RNA isolation from hydrogel-embedded cells was performed as follows: First, the hydrogel was dissolved using 80 mM EDTA (pH 7.4) in diethylpyrocarbonate (DEPC, Sigma-Aldrich)-treated water. Hydrogels were cut and mixed with 10 mL of EDTA in a conical tube. It was shaken vigorously in a vortex for 1 min and incubated at 37 °C for 10–20 min or until complete hydrogel dissolution. Every 5–8 min, it was shaken under the same conditions. Once the hydrogel was dissolved completely, the tubes were centrifuged at 9000× *g* for 5 min. The supernatant was discarded, and the pellet was resuspended in 750 μL of TRIsure and homogenized by pipetting until complete dissolution. Then, it was incubated at room temperature for 5 min and transferred to a microtube. It was centrifuged at 12,000× *g* for 15 min at 4 °C. The supernatant was transferred to a new microtube, and total RNA extraction proceeded following the TRIsure manufacturer’s recommendations. The RNA isolated was resuspended in DEPC-treated water. Then, RNA was cleaned up with AllPrep RNeasy columns (QIAGEN) following the manufacturer’s instructions. The concentration and purity of the RNA samples were analyzed in a NanoDrop, and the integrity was verified by an agarose gel (1% in TAE). Only RNA quality samples (with three bands corresponding to 5S, 18S, and 28S RNA subunits) were used for posterior retro-transcription reactions. 

#### 2.7.2. Retrotranscription of Total RNA from Embedded Cells

Following supplier instructions, a reverse transcription reaction was performed using the M-MLV reverse transcriptase enzyme (Invitrogen, Carlsbad, CA, USA). Random primers (Invitrogen) were used to obtain the transcription of all RNA. PCR was performed using the GoTaq polymerase enzyme (Promega, Maddison, WI, USA). The amount of cDNA was normalized in all samples. The oligonucleotides used are shown in Table 1. PCR products were analyzed with Image Lab software (Version 6.1) after electrophoresis with a 2% agarose gel. 

### 2.8. Trastuzumab-Fluorescein Conjugate Penetration in the Hydrogel with Embedded Cells

Trastuzumab, a commercial monoclonal antibody, was conjugated with fluorescein using 1-ethyl-3-(3-dimethyl aminopropyl) carbodiimide hydrochloride (EDC) as follows: In a microtube, a volume of 40 μL of Trastuzumab (Roche) with an initial concentration of 50 mg/mL solution was mixed with 960 μL of 0.1 M MES buffer (pH 4.5–5). Then, 20 μL of a freshly prepared 1-ethyl-3-(3-dimethyl aminopropyl) carbodiimide (EDC) hydrochloride solution (1 mg/mL) was added. It was homogenized, and 10 μL of sodium fluorescein solution (10 mg/mL, Sigma-Aldrich) was added and light-protected. The mixture was incubated for 2 h with gentle shaking at room temperature. The mixture was transferred to an Amicon tube (10 kDa, Merck Millipore) and ultrafiltrated, following the manufacturer’s instructions. The Trastuzumab-fluorescein conjugate was recovered, and aliquots were made and stored at 4 °C protected from light.

The detection of Trastuzumab-fluorescein conjugate in the hydrogels was evaluated in the encapsulated cells with confocal microscopy. The hydrogels were incubated with three Trastuzumab-fluorescein conjugates at different concentrations in a culture medium (5, 10, and 20 μg/mL) at 37 °C for 48 h under standard conditions. After incubation, hydrogels were cut with a cryostat, as described in Section 2.6. The cryosections were stained with Hoechst (nuclei) and analyzed by confocal microscopy to determine if the antibody penetrated the hydrogels.

### 2.9. Statistical Analysis

All statistical analyses were performed using Prism 10.0 (GraphPad Software, Inc., San Diego, CA, USA). Data were expressed as the mean ± standard error of the mean (SEM). For Alamar blue cell viability and metabolism assays, differences between multiple groups were evaluated by a one-way analysis of variance (ANOVA) followed by a Tukey’s test. In all statistical comparisons, differences were deemed significant when *p* values < 0.05.

## 3. Results and Discussion

### 3.1. Hydrogel Fabrication

**Figure 2:**
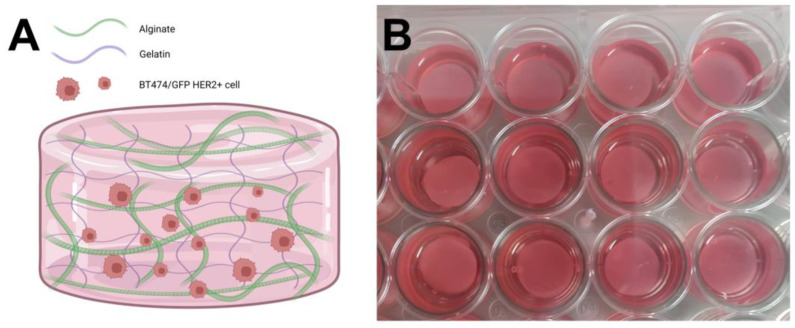
Hydrogels obtained. (**A**) Schematic representation of cells encapsulated in the hydrogels. (**B**) Photograph of the hydrogels with cells in 24-well plates.

With the described method, we obtained hydrogels with a final composition of 7.5% gelatin and 3.75% alginate cross-linked with CaCl_2_ (Figure 2). The hydrogels could remain without macroscopic fractures for up to 22 days.

### 3.2. Hydrogel Swelling Capacity

One of the features of hydrogels is their capacity to swell, retaining water within their structure without dissolving [17]. The swelling capacity affects the hydrogel’s physical features, such as microstructure and mechanical resistance [18]. 

**Figure 3:**
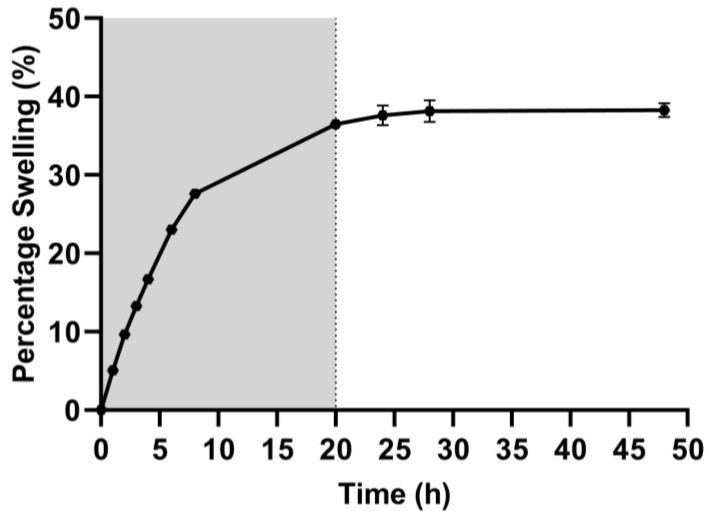
Hydrogel percentage swelling plot. The maximum percentage of swelling (40%) is reached after 20 h. The plot includes the mean and standard deviation. *n* = 3.

Figure 3 shows hydrogel swelling behavior from 0 to 50 h. During the first 8 h, the swelling percentage increases rapidly from 0 to 26% and gradually decreases until reaching swelling equilibrium at approximately 20 h. The maximum percentage of swelling was 38% of the hydrogel weight. This behavior of reaching equilibrium after 20 h has also been observed in other gelatin-based hydrogels [19].

Wang et al. reported that alginate fibers have a lower water uptake than alginate/gelatin fibers. In addition, higher gelatin concentrations increase the percentage of water absorption. However, at very high concentrations of gelatin, the water absorption can fracture the structure. Those results suggest that an optimal amount of gelatin improves hydrogel swelling properties [20]. 

Ye et al. made hybrid hydrogels of 6% gelatin and 1% alginate (cross-linked with transglutaminase and CaCl_2_). They evaluated the swelling capacity at 24 h and obtained an absorption rate of 20%. Wang et al. and Ye et al. observed that hydrogels containing only polysaccharides, such as alginate or chitosan, had a significantly lower swelling rate (4–1.5%). At the same time, hydrogels containing only gelatin had the highest rates. Gelatin–chitosan hydrogels had a lower swelling rate than gelatin–alginate hydrogels, possibly because chitosan molecules could contribute to developing a more compact hydrogel microstructure. In contrast, alginate molecules could have little effect on the microstructure [21].

The swelling capacity determined for the alginate–gelatin hydrogels is consistent, even in some cases higher than those reported for similar hydrogels. The swelling capacity improves the viability and proliferation of cells because it facilitates the transport of nutrients and metabolites, and it is considered that the swelling behavior of the alginate–gelatin hydrogel developed is suitable for embedded cells and favors cell survival.

### 3.3. Fourier-Transform Infrared Spectroscopy (FTIR)

**Figure 4:**
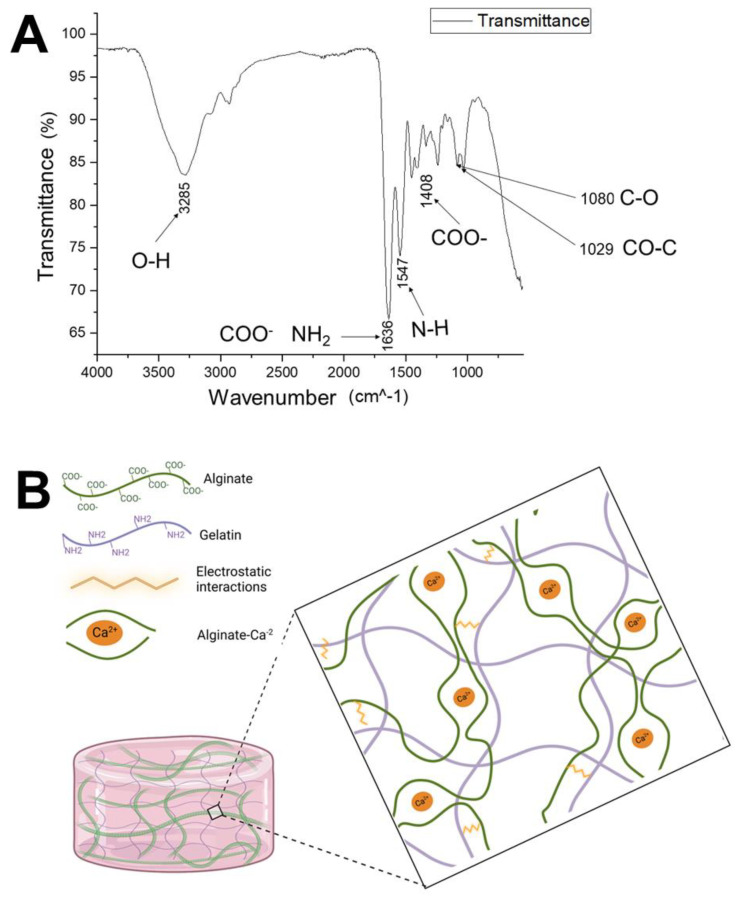
(**A**) Fourier-transform infrared spectroscopy (FTIR) spectrum of the gelatin–alginate hydrogels. The peak observed at 1636 $cm^{-1}$ could indicate electrostatic interactions between the gelatin and alginate. The peak at 1547 $cm^{-1}$ corresponds to the amide II group of the gelatin, while the peaks at 1080 and 1029 $cm^{-1}$ correspond to the mannuronic and guluronic units of the alginate. The peak at 3285 $cm^{-1}$ corresponds to the water held in the hydrogel. (**B**) Diagram of the possible network structure of the gelatin–alginate hydrogels. The alginate, cross-linked with calcium ions, forms the egg-box structure. Amino groups of gelatin amino acid residues form electrostatic interactions with the carboxyl groups of the alginate monomers.

Fourier-transform infrared (FTIR) spectroscopy analysis was performed with hydrogels prepared 48 h before. FTIR analysis permits the identification of the distinguishing functional groups of each polymer present in the hydrogel and knowing if there are interactions between them. Figure 4A shows the spectra obtained. Based on published data [22,23], the absorption bands were assigned to the vibrations of the corresponding functional groups.

The peak at 3285 $cm^{-1}$ corresponds to the vibration of O-H groups due to the large amount of water absorbed by the hydrogels. The peak at 1636 $cm^{-1}$ corresponds to the coupling of two absorption bands.

The one at 1616 $cm^{-1}$ is assigned to the asymmetric stretching vibration of the COO-, a group of sodium alginate, and the band at 1653 $cm^{-1}$ which corresponds to the amide I group, specifically gelatin’s N–H stretching vibration. This type of shift to a lower wavenumber (from 1653 to 1636 $cm^{-1}$) has been reported in gelatin–alginate hydrogels when polysaccharide content is increased [22] and indicates that the negative charge group of the alginate could interact electrostatically with the positive charge of the gelatin. Gelatin at a pH below its isoelectric point could form a complex with anionic polysaccharides, such as alginate, through electrostatic interactions, forming a polyelectrolyte complex. Type A gelatin has an isoelectric point between 8 and 9, and at a neutral pH culture medium (pH = 7), it would be in its cationic form [23].

In the same way, the peak 1408 $cm^{-1}$ corresponds to the displacement of the absorption band at 1418 $cm^{-1}$ assigned to the symmetric stretching vibration of COO- when the gelatin content is increased in hybrid gelatin–alginate hydrogels [22]. The spectrum shows characteristic absorption bands for protein structures. The peak at 1547 $cm^{-1}$ corresponds to the N–H stretching vibration of the amide II gelatin group [22]. Also, the spectrum shows characteristic bands of the polysaccharide structure due to the presence of alginate. The peaks at 1080 and 1029 $cm^{-1}$ correspond to the vibration of the C-O and CO-C groups in mannuronic and guluronic alginate units.

The positive charge of gelatin is because of the presence of arginine, lysine, hydroxylysine, and histidine residues in its structure [22]. Specifically, arginine is one of the principal amino acids in gelatin [24]. The negative charge of alginate is due to the carboxyl groups of the mannuronic and guluronic acid monomers. Gelatin–alginate solutions are stabilized by intermolecular electrostatic interactions between the alginate’s carboxyl groups and the gelatin’s amino groups [22]. 

The hydrogel’s FTIR spectrum shows that electrostatic interactions between gelatin and alginate conserve the distinctive functional groups of each polymer. The interactions between gelatin and alginate contribute to maintaining the hydrogel’s stability and influence in the microstructure since new nodules (junctions) can be generated in the red-shaped structure that forms the biopolymers. From these results, we can hypothesize the structure of the hydrogel, in which the alginate fibers are cross-linked by calcium ions, forming the egg-box structure. In addition, the amino groups of the gelatin interact with the carboxyl groups of the alginate through electrostatic interactions (Figure 4B).

### 3.4. Hydrogel Microstructure and Pore Size

The biocompatibility of a hydrogel is influenced by its microstructure, which affects the mechanical properties of the hydrogel and its ability to allow the diffusion of molecules [25]. To analyze the microstructure of the gelatin–alginate hydrogel, scanning electron microscopy (SEM) was conducted. Both cell-encapsulated and cell-free hydrogels were examined. 

**Figure 5:**
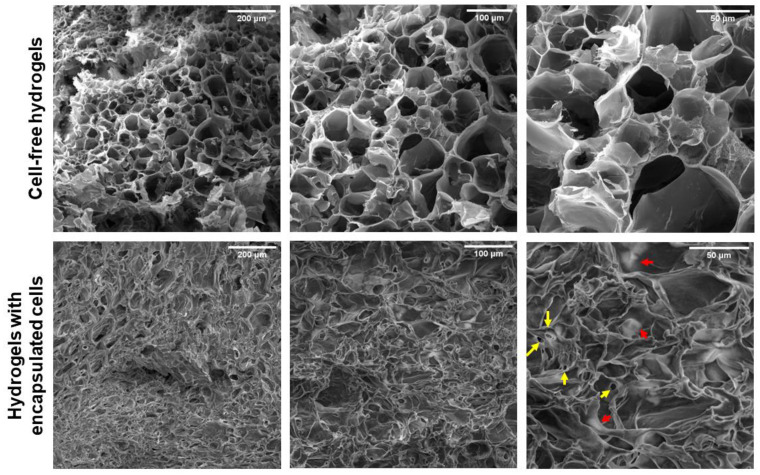
Hydrogel images obtained by SEM. Above, alginate–gelatin hydrogels without cells present a homogeneous porous structure (diameters between 20 and 150 μm) at 200×, 376×, and 890× magnification (digital zoom of the same field). Below, hydrogels with BT-474/GFP cells show an irregular structure. The yellow arrows indicate pores occupied by cells, and the red arrows indicate the presence of cells. The incubation time of the hydrogels was 48 h. The scale bar corresponds to 200, 100, and 50 µm.

The micrographs (Figure 5) show that the gelatin–alginate hydrogels exhibit an interconnected, homogeneous, and porous internal structure like a honeycomb with an average of 45 μm diameter (20 to 125 μm range). These values are consistent with what has been reported for gelatin–chitosan hybrid hydrogels with pores between 15 and 40 μm [26], as well as in gelatin hydrogels cross-linked with metal ions [27] and in alginate hydrogels cross-linked with calcium [28,29]. The interconnected pores in hydrogels serve as channels that allow greater water uptake and faster nutrient diffusion within the hydrogel [28]. In addition, it favors the diffusion of signaling molecules and metabolite elimination, allowing cell progression and migration [30]. Hydrogel pores without cells range in diameter from 20 to 125 μm. 

The interconnected pores in hydrogels serve as channels that allow greater water uptake and faster nutrient diffusion within the hydrogel [28]. In addition, it has been reported that this size range is suitable for nutrient and oxygen penetration and the diffusion of cellular waste [30]. 

For regenerative medicine, it is reported that when the scaffolds have pore sizes greater than 50–100 μm, they lose their mechanical and hydrodynamic properties [31]. Additionally, it has been reported that the presence of pores >200 μm could cause cells to spread out of the hydrogel [32]. When the pore size is small (<200 μm), cells can form adhesions in three dimensions, whereas larger pores provide fewer opportunities for cell adhesion and only two-dimensional adhesion can be generated. Therefore, the pore size influences the cell phenotype [33]. Therefore, a pore diameter of 45–65 μm could be adequate for cell growth because it allows the cells to be embedded in a three-dimensional structure without impairing the mechanical properties of the hydrogel. On the other hand, in hydrogels with encapsulated cells, the interconnected pore structure observed in cell-free hydrogels is not appreciated (Figure 5). 

**Figure 6:**
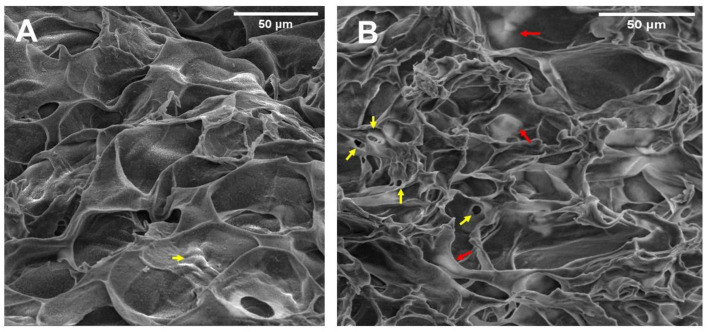
BT-474/GFP cells into the hydrogels observed by SEM. (**A**) The yellow arrow indicates a pair of cells below the hydrogel. (**B**) The yellow arrows show cell-occupied pores, and the red arrows indicate cells’ presence (visualize as white shadows). Due to the cells being added prior to cross-linking, the polymers gelled around the cell structures. Hydrogels had 48 h of incubation. The images correspond to 1000× magnification. The scale bar corresponds to 50 µm.

Figure 6 shows BT-474/GFP cells encapsulated in the gelatin–alginate hydrogel. The yellow arrows highlight cells below the polymer network (Figure 5A). Additionally, the pore size decreased at 4–20 μm due to cells being added to the biopolymer mix before cross-linking, possibly because polymers gelled around cells, creating those smaller pores. Modifications in hydrogel microstructure due to the presence of cells were previously observed by Thorpe et al. in hydroxyapatite hydrogels with human mesenchymal stem cells (hMSC) added [31]. Red arrows correspond to cells adhering to the hydrogel (Figure 5B). These images show that BT-474/GFP cells are contained within the hydrogel structure (they do not sediment to the bottom). In addition, cells present a three-dimensional morphology that is not observed in monolayer cultures. 

### 3.5. Hydrogel Biocompatibility 

**Figure 7:**
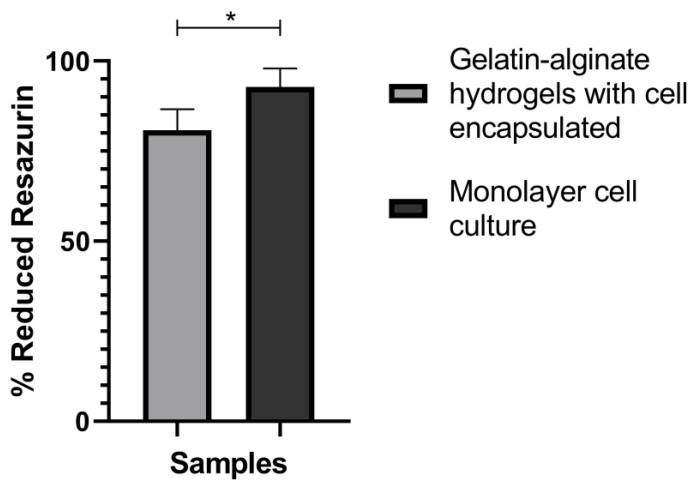
Gelatin–alginate hydrogels biocompatibility after 24 h. Percentage of reduced resazurin plot, an ANOVA test was performed with a confidence level of 95%. * *p* < 0.05. *n* = 3.

Hydrogel biocompatibility was evaluated by resazurin reduction. Figure 7 shows the results of the biocompatibility test. Cells encapsulated for 24 h in the gelatin–alginate hydrogel have a metabolic activity of ~90% compared to cells in a monolayer, indicating that this hydrogel is biocompatible with cells. Also, it was considered that the hydrogel, the fabrication procedure, and the cross-linking process did not affect cell viability.

### 3.6. Morphology and Distribution of Living Embedded Cells within Hydrogel

**Figure 8:**
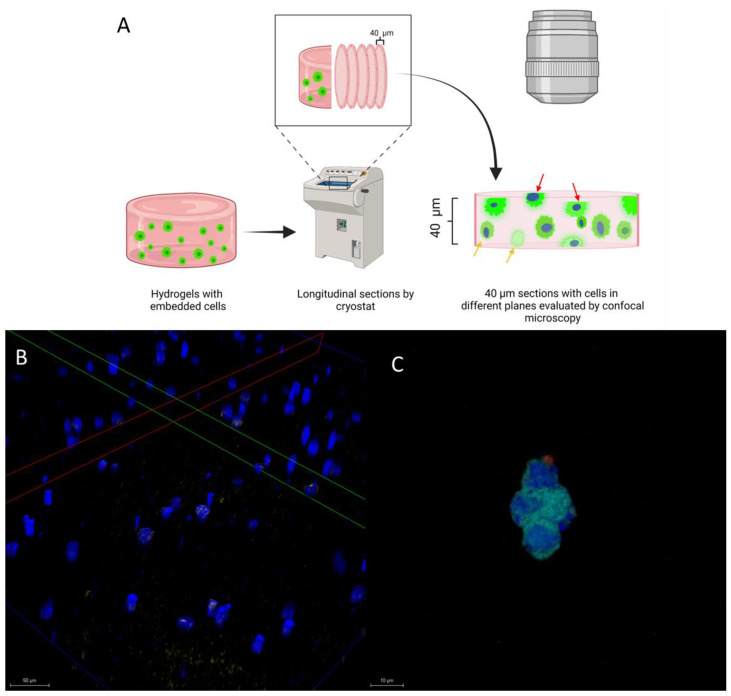
Cell distribution within the hydrogel after 48 h of incubation. (**A**) Schematic workflow of 40 μm cryosections for confocal analysis. (**B**) The Z axis of the BT-474/GFP cells’ projection shows different focal planes (according to the red and green reference lines) within the hydrogel. The upper cell is found in focal planes closest to the 10× objective, while the least intense cells (bottom) are found in focal planes far from the microscope objective, indicating dispersed cell distribution along the hydrogel thickness. Projections obtained by confocal microscopy at 10×, (**C**) Image of embedded BT-474/GFP cells with auto-aggregation indicating tropism between cells. Imagine obtaining it with a 40× objective. Cells express GFP (green), and Hoechst (blue) nuclei are stained. The scale bar corresponds to 50 μm or 10 μm.

Cryosections were analyzed by confocal microscopy. A schematic workflow for confocal microscopy analysis is shown in Figure 8A, based on the BT-474 cells expressing GFP and the nuclei stained with Hoechst. In a 40 μm-thick section, embedded cells can be found at different heights within the hydrogel. 

Cells observed in Figure 8B show an oblique projection in the *Z* axis (red and green lines) across the 40 μm sample section, indicating that each cell has a different arrangement in space, implying that the cell is growing between the pores of the hydrogel structure. 

Figure 8C shows an image of a group of encapsulated cells in direct contact obtained by confocal microscopy. The images of Figure 7, together with those observed by SEM (Figure 4 and Figure 5), show that embedded BT-474/GFP cells present a three-dimensional conformation. This feature cannot be imitated in monolayer cultures, where cells adhere to the same plane.

### 3.7. Metabolic Activity of Embedded Cell

**Figure 9:**
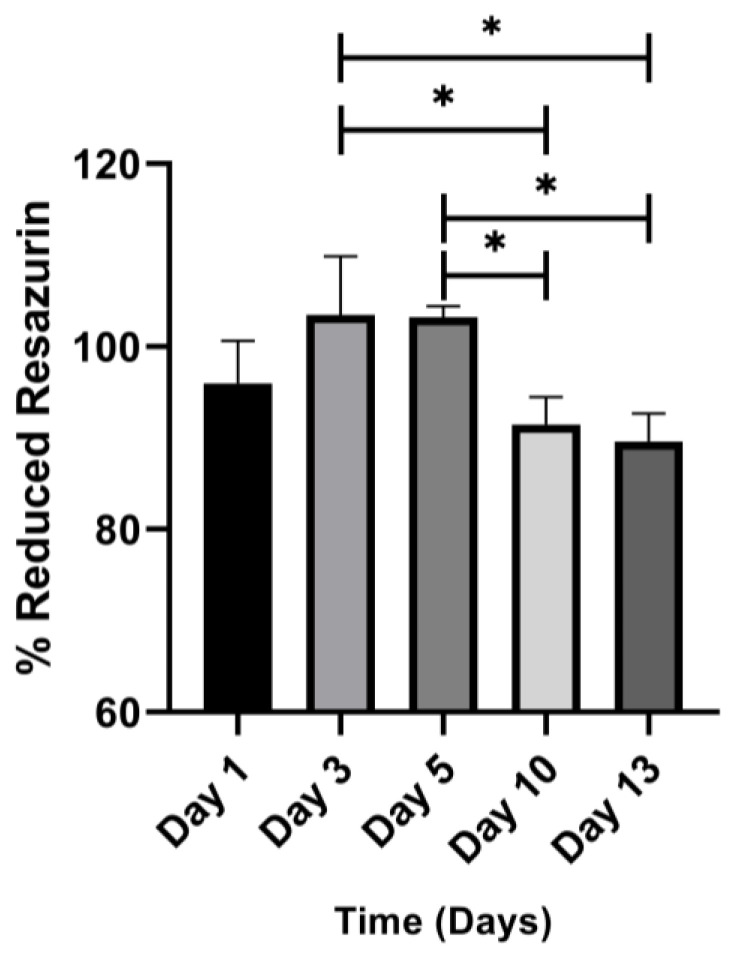
Metabolic activity results. No significant differences are observed between the percentage of reduced resazurin on day 1 and day 10 or 13; this could relate to the fact that encapsulated cells are in a state of equilibrium between cell proliferation and death, like what is observed in a dormant tumor. ANOVA statistical test was performed with a confidence level of 95% (* *p* < 0.05). *n* = 3.

The metabolic activity was measured by the percentage of resazurin reduced on days 1, 3, 5, 10, and 13 after encapsulation (Figure 9). On the first day (24 h after encapsulating the cells), the percentage of resazurin was 96%, indicating that the process of production and cross-linking of the hydrogel is not cytotoxic for BT-474/GFP cells. On days 3 and 5, the resazurin to resorufin reduction percentage was 103%. However, this result was not statistically significant (*p* = 0.23 and *p* = 0.26) compared to day 1. Ten days after encapsulation, cells were still viable. The resazurin reduction percentage was 91.5%, significantly different from the reduction percentages on days 3 (*p* = 0.030) and 5 (*p* = 0.033). However, there was no difference in the percentage reduction on day 1 (*p* = 0.66). On day 13, the cells were still viable, with a reduced resazurin percentage of 89.6%. When comparing days, there was a significant difference compared to the percentage of reduced resazurin corresponding to days 3 (*p* = 0.012) and 5 (*p* = 0.014), but there was no difference with the reduction percentage for day 1; this is related to the number of viable cells maintained during incubation (Figure 9).

Farino et al. reported that in synthetic polymer hydrogels, MDA-MB-231 triple-negative breast cancer cells did not form cell aggregates but were distributed individually throughout the hydrogel. These hydrogels were characterized by high cell death and low metabolic activity [34]. Also, Pradhan and Slater reported hydrogels with similar features. The authors defined this type of cell as being in a state of cellular latency characterized by low proliferation without a statistically significant increase in metabolic activity [35]. These hydrogels recapitulate the characteristics of disseminated tumor cells (DTC) that, when infiltrating secondary organs, can acquire a latent state before forming new tumors [35]. These states are also observed before forming a primary tumor and in local cancer recurrence. Our gelatin–alginate hydrogels have characteristics like those observed by Pradhan and Slater, where BT-474/GFP cells do not form massive cell aggregates (spheroid-like, Figure 7). In contrast, they are found in small aggregations formed by a few cells (Figure 7) or individually. Although metabolic activity is at 80%, there is no significant difference between metabolic activity on day 1 and days 10 or 13. 

### 3.8. Evaluation of Genes Related to Hypoxia, Apoptosis, and HER2 Receptor 

**Figure 10:**
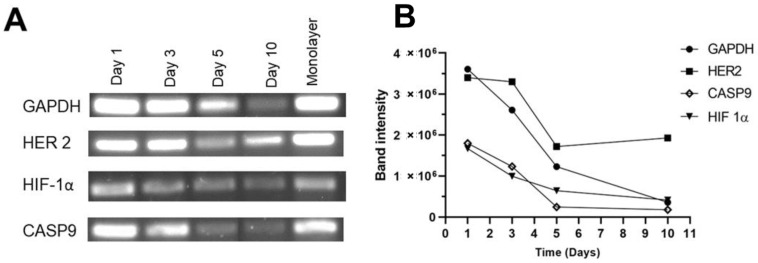
Results of the amplification of *GAPDH, HER2, HIF1-α*, and *Caspase 9* genes. (**A**) Electrophoresis gel with gene amplifications at days 1, 3, 5, and 10 of incubation and monolayer amplification at day 1. (**B**) Plot showing amplifications. At day 10, *GAPDH, HIF1-α*, and *Caspase 9* were amplified at the same level, while *HER2* was amplified higher.

Total RNA was extracted from cells that were encapsulated within the hydrogel for 1, 3, 5, and 10 days. An RT was carried out, followed by an endpoint PCR using 90 ng of cDNA, and agarose gels were run for each gene (Figure 10A). The Image Lab software was used to analyze the agarose gels. The plot in Figure 9 shows the behavior of each gene (*GAPDH, HER2, CASP9*, and *HIF1-α*). Apparently, on day 10, there was an increase in *HER2*, while *GAPDH*, *CASP9*, and *HIF1-α* decreased over time.

The enzyme glyceraldehyde 3-phosphate dehydrogenase (GAPDH) is one of the 10 enzymes that catalyze reactions in glycolysis. Specifically, GAPDH catalyzes the conversion of glyceraldehyde-3-phosphate to glyceraldehyde-1,3-bisphosphate with the participation of NAD. GADPH is widely used as a reference gene in Western blot and q-PCR analyses. It has been reported that GAPDH expression is affected by nutritional cell conditions [36]. According to the results, the amplification of *GAPDH* in BT-474/GFP cells embedded in the hydrogel decreases during the encapsulation time. The metabolism may decrease, or other routes for energy generation are used than glycolysis; this coincides with the results of Carcereri et al. in MDA-MB-231 breast cancer cells studied in latency, where they found that glucose consumption was reduced in these cells and, therefore, the gene expression of GADPH was also decreased [37]. Some studies suggest that cancer cells have heterogeneous and flexible metabolic preferences that depend on intrinsic factors (such as mutations and genetic variations) and microenvironmental factors, for example, interactions with the ECM. Slow-proliferating tumor cells are, in most cases, dependent on mitochondrial respiration [38].

Hypoxia-inducible factor 1 (HIF-1α) regulates oxygen homeostasis. The proteasome degrades the HIF-1α subunit under normoxic conditions. However, it translocates to the nucleus under hypoxic conditions and forms a heterodimer with HIF-1β. In this way, they activate the transcription of genes that mediate energy metabolism, erythropoiesis, and vascularization in response to hypoxia [39]. In cancer, these mechanisms favor vascularization, growth, cell-matrix remodeling, cell mobility, local invasion, and metastasis. The oxygen level in a tumor depends on the initial oxygenation of the tissue, its size, and the stage of the tumor. Normoxia in healthy human tissues ranges from 9.5% O_2_ in the renal cortex to 4.6% O_2_ in the brain. In the case of the breast, the oxygen concentration considered normoxic is 8.5%. A state of hypoxia is considered when oxygenation is below the expected concentration of the tissue and, on average, varies between 1% and 2% O_2_. In the case of the breast, concentrations equal to or less than 1.5% of O_2_ are considered hypoxic [40]. HIF overexpression in breast cancer is considered an unfavorable feature [41]. The results obtained in alginate–gelatin hydrogels showed that the amplification of HIF-1α decreased over encapsulation time (Figure 10). Fluegen et al. reported that the hypoxic microenvironment in primary tumors activates latency programs [42]. 

Caspases are proteases that mediate programmed cell death and inflammation. Specifically, caspase 9 (CASP9) is an initiator enzyme that activates because of the permeabilization of the mitochondrial outer membrane. Once activated, caspase-9 can cleave and activate caspase-3 and caspase-7 (effector caspases) [43]. In addition to initiating apoptosis, its function in non-apoptotic cell death processes and autophagy has been documented [44]. The results in Figure 9 show that CASP9 decreases in the initial five days and then remains constant; this could indicate that the mitochondria of embedded cells remain intact. Furthermore, if there is cell death, it could be caused by non-apoptotic processes. In contrast, in other 3D cultures of breast cancer, CASP9 and CASP3 have been reported to be overexpressed [45].

Overexpression of the HER2 receptor is associated with excessive cell division, leading to more aggressive tumors with a higher risk of metastasis. The HER2 receptor is used as a target molecule for targeted therapies, and therefore it is essential to have 3D models that maintain HER2 expression for in vitro evaluation of new therapeutic molecules. It has been observed that in 3D cultures, there is an increase in the expression of the HER2 receptor compared to monolayer cultures. Azimi et al. seeded tumoroids from SKBR3 HER2-positive breast cancer cells on type I collagen hydrogels and observed an increase in HER2 receptor levels [45]. Similarly, Cheng et al. observed that non-malignant breast cells, MCF10A, overexpressed HER2 when cultured on Matrigel [46]. According to our results, the encapsulated BT-474/GFP cells are in a low proliferation state correlated with the resazurin reduction assay and the *GAPDH* gene amplification; however, the *HER2* gene amplification remains certainly lower than days 1 and 3, but in comparison with *GAPDH* and the other genes, the *HER2* gene presents a higher band intensity (Figure 10B). Tumoral cells like BT-474 could use this receptor to promote proliferation when optimal metabolic conditions are present. Furthermore, this feature makes the developed hydrogel suitable for evaluating HER2 interactions with biomolecules or even latency phenomena.

### 3.9. Trastuzumab-Fluorescein Conjugate Penetration into the Hydrogel

**Figure 11:**
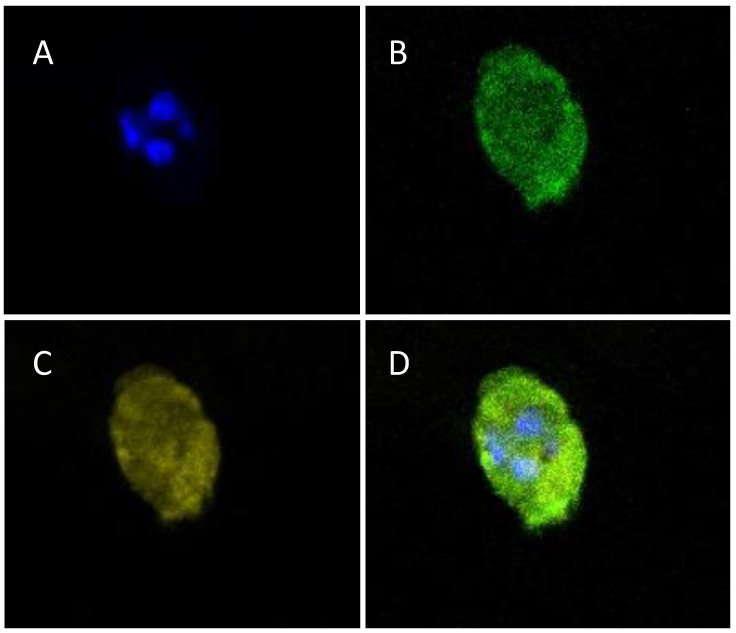
Interaction of the Trastuzumab-fluorescein conjugate with BT-474/GFP cells encapsulated in the gelatin–alginate hydrogel after 48 h of incubation. Representative images of embedded cell interactions in the hydrogel. (**A**) Hoechst-stained nuclei (blue); (**B**) Cell cytoplasm with an autologous expression of GFP protein; (**C**) Trastuzumab-fluorescein used at 10 μg/mL (assigned in yellow) is detected all around the cell’s membrane; (**D**) Merge of all signals. The Trastuzumab-fluorescein conjugate penetrates inside the hydrogel due to the pore size. Also, the BT-474/GFP cells maintain HER2 expression inside the hydrogel, as demonstrated by Trastuzumab-fluorescein detection. All images correspond to 40× confocal microscopy. The scale bar corresponds to 10 μm.

The Trastuzumab-fluorescein conjugate was in contact with the encapsulated cells for 48 h to evaluate the monoclonal antibody penetration into the hydrogel. The results are shown in Figure 11, and the conjugate was able to diffuse through the hydrogel and interact with the encapsulated cells regardless of the conjugate concentration used; this coincides with the results of the physicochemical hydrogel characterization, in which it was observed that the size of the pores was between 20 and 125 μm. This size allows the diffusion of nutrients in the same way that it allows the diffusion of IgG-type antibodies and could allow the penetration of smaller therapeutic biomolecules. Additionally, it would allow encapsulated cells to be analyzed by immunofluorescence assays.

Due to their diffusion properties and the fact that encapsulated cells maintain HER2 receptor expression, these hydrogels could help assess the binding capacity and cellular responses of new anti-HER2 therapies.

## 4. Conclusions

In this study, a hybrid gelatin–alginate hydrogel biocompatible with living HER2+ breast cancer cells was developed. The hydrogel’s physicochemical features allowed the diffusion of nutrients and permitted embedded cells within the hydrogel.

Embedded cells were distributed individually throughout the hydrogel. They also maintained the expression of the HER2 receptor; the transcription factor HIF-1α was expressed; the expression of CASP9 remained constant; and the expression of GADPDH decreased. These phenomena could be because embedded cells have little cellular activity but express the HER2 receptor, which promotes cell proliferation in an optimal environment. 

Trastuzumab-fluorescein conjugate diffused into the hydrogel and bonded to the target receptor. This hydrogel could help evaluate HER2 receptor-targeted therapies in BT-474/GFP cells in a three-dimensional environment.

## Figures and Tables

**Table 1:** Oligonucleotide sequence used in PCR for hydrogel-encapsulated cells and monolayer.

Gene	Oligonucleotide Sequence 5′-3′	Tm (°C)
*HER 2*	FW: GGAAGTACACGATGCGGAGACT	66.8
	RV: ACCTTCCTCAGCTCCGTCTCTT	66.9
*HIF-1α*	FW: TATGAGCCAGAAGAACTTTTAGGC	63.8
	RV: CACCTCTTTTGGCAAGCATCCTG	70.9
*GADPH*	FW: GCACAGTCAAGGCCGAGAAT	65.7
	RV: GCCTTCTCCATGGTGGTGAA	65.3
*CASP 9*	FW: GTTTGAGGACCTTCGACCAGCT	68
	RV: CAACGTACCAGGAGCCACTCTT	66.7

## Data Availability

Data are contained within the article.
